# Methods for drug safety signal detection using routinely collected observational electronic health care data: A systematic review

**DOI:** 10.1002/pds.5548

**Published:** 2022-11-02

**Authors:** Astrid Coste, Angel Wong, Marleen Bokern, Andrew Bate, Ian J. Douglas

**Affiliations:** ^1^ Department of Non‐Communicable Disease Epidemiology LSHTM London UK; ^2^ Global Safety GSK Brentford UK

**Keywords:** drug safety surveillance, pharmacoepidemiology, pharmacovigilance, real world data, signal detection, systematic review

## Abstract

**Purpose:**

Signal detection is a crucial step in the discovery of post‐marketing adverse drug reactions. There is a growing interest in using routinely collected data to complement established spontaneous report analyses. This work aims to systematically review the methods for drug safety signal detection using routinely collected healthcare data and their performance, both in general and for specific types of drugs and outcomes.

**Methods:**

We conducted a systematic review following the PRISMA guidelines, and registered a protocol in PROSPERO. MEDLINE, EMBASE, PubMed, Web of Science, Scopus, and the Cochrane Library were searched until July 13, 2021.

**Results:**

The review included 101 articles, among which there were 39 methodological works, 25 performance assessment papers, and 24 observational studies. Methods included adaptations from those used with spontaneous reports, traditional epidemiological designs, methods specific to signal detection with real‐world data. More recently, implementations of machine learning have been studied in the literature. Twenty‐five studies evaluated method performances, 16 of them using the area under the curve (AUC) for a range of positive and negative controls as their main measure. Despite the likelihood that performance measurement could vary by drug‐event pair, only 10 studies reported performance stratified by drugs and outcomes, in a heterogeneous manner. The replicability of the performance assessment results was limited due to lack of transparency in reporting and the lack of a gold standard reference set.

**Conclusions:**

A variety of methods have been described in the literature for signal detection with routinely collected data. No method showed superior performance in all papers and across all drugs and outcomes, performance assessment and reporting were heterogeneous. However, there is limited evidence that self‐controlled designs, high dimensional propensity scores, and machine learning can achieve higher performances than other methods.

**Key Points**

- There has been a growing interest in the last 15 years to use routinely collected data to complement spontaneous reports for drug safety signal detection.
- This is the first systematic review including 101 studies, which quantified the use of a wide variety of methods for drug safety signal detection with routinely collected data and assessed their comparative performance. While self‐controlled methods performed overall well there were no direct comparisons of all approaches in the 25 performance assessment studies.
- Transparency, replicability and, due in part to the lack of a gold standard reference set, comparability between studies was limited.
- Although the suitability of epidemiological methods varies by nature of exposure and outcome, stratified performance was only available in 9.9% of studies, adding difficulty to the identification of useful methods for signal detection.

## 1 INTRODUCTION

Signal detection is the process of identifying emerging true associations as early as possible, ideally leading to further action while effectively avoiding false positives. For decades, spontaneous reports (SRs) have been the primary approach for detecting adverse drug reactions (ADRs) not picked up in clinical trials,^1^ and remain so despite their well‐recognized limitations.^2^, ^3^ There is a growing interest in using real‐world data (RWD), including claims data and electronic health records (EHRs). Their potential for signal detection has been recognized as a hope for potentially faster and more efficient post marketing surveillance.^4^ Several initiatives have provided methodological input for drug safety signal detection using RWD^5^, ^6^ and have evaluated the performance of various methods against a set of positive and negative controls.

Methods for signal detection with RWD were reviewed by Arnaud et al.^7^ until 2016, focusing on both their overall performance regardless of types of drugs and outcomes and secondly understandability by stakeholders. However, epidemiological methods are differentially valid depending on the nature of the drug and outcome studied, and a single method applied to a wide range of drugs and outcomes without consideration of its optimal application could lead to poor detection^8^ It is therefore useful to explore whether this issue has been considered in signal detection, or whether a one fits all approach has been largely used for simplicity. Further, novel methods have also been developed since this review.^9^, ^10^

Therefore, this systematic review aimed to: (1) update the list of methods for drug safety signal detection using routinely collected data and quantify the extent of their published; (2) summarize and compare methods performance regarding ability to detect signals in routinely collected observational data; and (3) assess the performance of each method for specific types of exposures and outcomes.

## 2 METHODS

### 2.1 Search strategy

The systematic review was conducted following the protocol registered at PROSPERO (registration number CRD42021267610). We searched MEDLINE and EMBASE via OVID, Web of science, Scopus, PubMed, and the Cochrane Library with no restriction on the period on July 13, 2021.

Keywords and Medical Subject Headings (MeSH) based on (1) routinely collected data, (2) pharmacoepidemiology or drug safety. and (3) signal detection were used (Appendix S1). The reference lists from identified literature reviews were screened to identify additional works.

Included studies were (1) describing an epidemiological study design or statistical method for signal detection using routinely collected observational data; (2) evaluating their performance; or (3) applying these methods to screen drug‐outcome pairs. We excluded studies relying on free‐text data because methods mainly rely on natural language processing which are different to that used for structured data^11^ as well as conference abstracts. The original protocol was modified by not including vaccine related studies as methods for vaccine signal detection have their specific limitations and different considerations from other medications.^12^

Articles were firstly screened by title and abstracts, followed by a full‐text evaluation for eligible papers. A second reviewer assessed all the included publications and a sample of the excluded ones. Any disagreement was resolved by discussion.

### 2.2 Data extraction

We extracted data based on the RECORD Pharmacoepidemiology Checklist,^13^ focusing on the details of the methods: design, statistical outputs; exposure(s), outcome(s), results, and performances of the methods.

The risks of bias and confounding, the appropriateness of the ADR testing and the degree to which the database captures outcomes were also assessed.

### 2.3 Data analysis

The characteristics of the included studies and the methods for drug safety signal detection were reported. Methods for drug safety signal detection using RWD were described and the number of times they were used was quantified. The performance of these methods was assessed using measures presented in the literature, both in general for all drug/outcome pairs and by drug and outcome when this was available.

## 3 RESULTS

### 3.1 Studies identified

**FIGURE 1:**
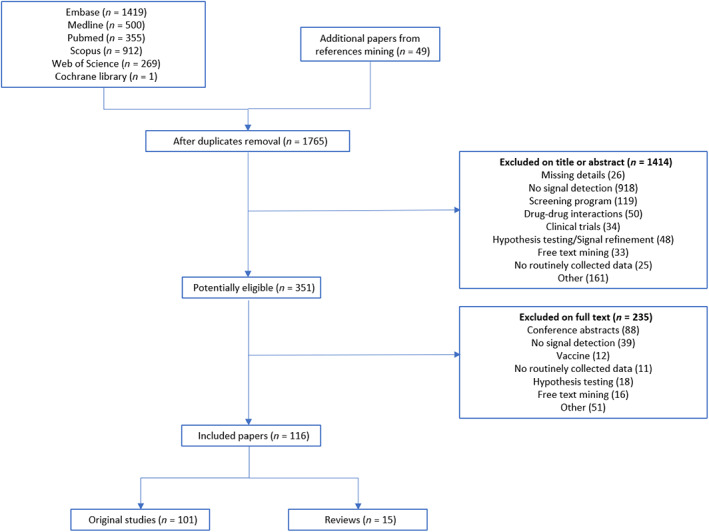
Flowchart of inclusion

We screened 1765 titles and abstracts. After applying inclusion and exclusion criteria, 351 papers were classified as potentially eligible (Figure 1). Of those, 116 relevant studies were included in the review, with 101 original studies and 15 reviews.

**FIGURE 2:**
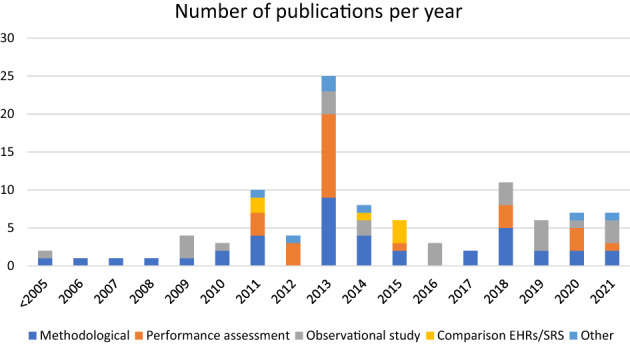
Number of studies by year. “Observational study” in the graph refers to the category “application of method without performance assessment” in Table 1.

Of the included studies, 38.6% purely described methods (Table 1), 24.8% were about performance assessment and 23.8% were observational studies without performance assessment. Among the studies, 5.9% of them compared the use of EHRs and SRs for signal detection.^1^, ^14^, ^15^, ^16^, ^17^, ^18^ The remaining 6.9% included a recent PhD thesis,^19^ two commentaries,^20^, ^21^ a study aiming to establish a reference standard for signal detection^22^ and 3 studies looking at the significance of signal detection results.^23^, ^24^, ^25^ Most studies (88.1%) used traditional EHRs or claims data, while 6.9% used abnormal laboratory results^9^, ^26^, ^27^, ^28^, ^29^, ^30^, ^31^ and a prescription only dataset (5%) where prescriptions are used as proxies for diagnoses.^32^, ^33^, ^34^, ^35^, ^36^ The aim of our systematic review was to identify original research and so any review articles we identified within scope were only used to provide potential further original research publications for inclusion and their contents were not extracted.^4^, ^7^, ^23^, ^37^, ^38^, ^39^, ^40^, ^41^, ^42^, ^43^, ^44^, ^45^, ^46^, ^47^ A third of the studies were published after 2016, year of the latest review on the topic, as shown on Figure 2.

**TABLE 1:** Summary characteristics of the publications included in the review

Characteristic	Number of publications
**Primary objective of the paper**	
Method description	39 (38.6%)
Performance assessment	25 (24.8%)
Data comparison between EHRs and SRs	6 (5.9%)
Application of method without performance assessment	24 (23.8%)
Other^a^	7 (6.9%)
**Location of data**	
United States	43 (42.6%)
Europe	37 (36.7%)
Asia/Australia	18 (17.8%)
International	3 (3.0%)
**Approach**	
Outcome based	40 (39.6%)
Exposure based	26 (25.7%)
Both drugs and outcomes specified	6 (5.9%)
All drugs and outcomes in the database(s)	4 (4.0%)
None (purely methodological)	25 (24.8%)
**Type of data used by the method**	
Method based on prescription and diagnoses codes	89 (88.1%)
Method based on prescription data only	5 (5.0%)
Method based on the comparison of laboratory test results	7 (6.9%)

^a^ Other = PhD thesis, commentaries, reference standard.

### 3.2 Quality assessment

There are no standard criteria to assess the quality of signal detection studies beyond general quality assessment tools and guidance for RWD studies. Often, the definitions for the chosen drugs and outcomes were not specified, and specific implementation in the databases was rarely specified. The codes and code lists were rarely made available. Notably, the Observational Medical Outcome Partnership (OMOP) initiative has now switched to the Observational Health Data Sciences and Informatics (OHDSI), so that previous OMOP reports are not publicly available on the website as of 1st June 1, 2022. This limits the reproducibility of some included studies. Other more recent studies published Supporting Information (Supplement S1 such as details on outcome definition or on performance results.^48^, ^49^

### 3.3 Methods for drug safety signal detection

A wide range of methods were described in the included studies, and are summarized in Tables 2 and 3 following a classification used by Arnaud et al.^7^ Overall, the literature focussed on adapting disproportionality analysis methods to signal detection and implementing traditional epidemiological designs. Other methods, using Bayesian network models, the Weibull shape parameter or likelihood ratio tests were proposed in methodological papers but used in a single or no observational study so are not included in the following tables.^102^, ^103^

**TABLE 2:** Number of times each method applied twice or more was used across the publications of the review

Method	Number of papers using the design^a^
**Disproportionality analysis**	
PRR	9 (17.3%)
ROR	8 (15.4%)
BCPNN	9 (17.3%)
GPS/MGPS	6 (11.5%)
LGPS/LEOPARD	12 (23.1%)
Other	8 (15.4%)
**Subtotal**	**52 (100.0%)**
**Traditional epidemiological designs**	
Self‐controlled case series	15 (34.1%)
Self‐controlled cohort	5 (11.4%)
New‐user cohort	5 (11.4%)
Case–control	13 (29.5%)
Case‐crossover	3 (6.8%)
Case‐population	3 (6.8%)
**Subtotal**	**44 (100.0%)**
**Temporal association**	
Temporal pattern discovery	10 (50.0%)
MUTARA/HUNT	6 (30.0%)
Fuzzy‐based logic **Subtotal**	4 (20.0%) **20 (100.0%)**
**Sequence symmetry analysis**	6 (100.0%)
**Sequential testing**	
MaxSPRT	4 (66.7%)
CSSP	2 (33.3%)
**Subtotal**	6 (100.0%)
**Tree‐based scan statistic**	9 (100.0%)
**Other designs including machine learning**	13 (100.0%)
**Lab results**	9 (100.0%)
**Prescription only methods**	5 (100.0%)

Abbreviations: BCPNN, Bayesian Confidence Propagation Neural Network; CSSP, Conditional Sequential Sampling Procedure; GPS, Gamma Poisson Shrinker; HUNT, Highlighting Unexpected TARs Neglecting TARs; LEOPARD, Longitudinal Evaluation of Observational Profiles of Adverse events Related to Drugs; LGPS, Longitudinal Gamma Poisson Shrinker; MaxSPRT, Maximized Sequential Probability Ratio Test; MGPS, Multi‐Item Gamma Poisson Shrinker; MUTARA, Mining Unexpected Temporal Association Rules (TARs) Given the Antecedent; PRR, Proportional Reporting Ratio; ROR, Reporting Odds Ratio.

^a^ Studies exploring more than one method were counted for each of the methods they considered, so that the total number of papers in this table does not correspond to the number of included studies.

**TABLE 3:** Overview of methods for drug safety signal detection using RWD mentioned or applied in more than one study as reported in the included papers

Method	Stated general concept	Reported advantages	Reported weaknesses	Additional comments
**Disproportionality analysis (DP)**				
PRR and ROR	Originally applied to SRs to determine the degree of disproportionality between the reporting of a condition for a given drug.^50^ Focus on a 2 × 2 contingency table to compare the observed number of records to an expected number of records.^51^, ^52^	Easy to calculate and to implement.	Unstable with small number of events and large confidence intervals leads to high false‐positive rates for rare events.^45^ Designed for cross sectional data, so the total number of cases available in EHR databases is not used.	
Information Component (BCPNN)	A fully Bayesian (predefined prior) disproportionality method using shrinkage of observed‐to‐expected (*O*/*E*) scores to calculate an Information Component (IC).^53^ Sometimes implemented in a neural network (BCPNN) where the IC is the weight.	Addresses the high false‐positive rate issue of the SR‐like methods by greater shrinkage when little data support reducing spurious chance findings.^50^	As above, in addition harder interpretability as impact of Bayesian shrinkage adds additional complexity to result interpretation.	
GPS/MGPS	GPS is another Bayesian method for shrinkage of the observed/expected ratio.^54^ MGPS is an extension for analysis of drug–drug‐event interactions^45^	Same as BCPNN, empirical shrinkage so shrinkage strength adapts to specific data set.	Primarily same as BCPNN, and impact of shrinkage variable. Computationally intensive.	
LGPS	Adaptation of the GPS method to longitudinal data.^53^ LGPS compares the incidence rate of outcome during exposure risk period to the background rate for all people.	Has been used in conjunction with the LEOPARD method, to theoretically handle protopathic and indication biases. LEOPARD compares the rate of prescription prior to the events to that after.^54^	Impact of Bayesian prior as above.	
**Traditional epidemiological designs**				
Self‐controlled case series (SCCS)	Comparison of the event rate during exposed and unexposed time within the same individual.^55^	Eliminate time invariant confounders, advantageous when baseline covariates not measured with sufficient precision. Cases only: computational savings.^55^	Accurate dating of outcomes is crucial, best applies to intermittent exposures and transient or acute events.^56^	Several further modifications have been proposed, but not yet implemented in signal detection studies.^10^, ^57^, ^58^
Self‐controlled cohort (SCC)	Comprising both a cohort and self‐controlled adjustment.	Utilizes an external control group to adjust for remaining time‐varying confounding after the self‐controlled component.^8^	Sensitive to differences between risk and control period specific to the exposed group, such as protopathic bias.^7^	
New user cohort (NUC)	Compares the rate of events in a cohort initiating the drug of interest versus a cohort not initiating this drug.^59^, ^60^	Broadly applicable method,^59^ active comparator approach to address confounding by indication.^64^ Computation of the absolute risk of events.^7^	Higher computational requirements than self‐controlled methods. Between person confounding. Need for a predefined comparator(s) as its appropriateness is difficult to assess in real‐world settings.	
Case–control (CC) method	Compares the frequency of exposure of “cases” who experienced the outcome with that of matched “controls” who did not experience the outcome.^61^	Effective for rare outcomes.^65^	Possibly challenging control selection, susceptible to between person confounding. High computational requirements, slowest method in OMOP.^66^	
Case‐crossover	Uses within‐person comparison of exposure in the case period compared to that in the control period.^62^	Similar to other self‐controlled designs.	Subject to bias when exposure time trend is present.^62^	
Case‐population	Similar to case–control design, but using the entire population as control group.^63^	Increased statistical precision compared to CC.^63^	Higher computational requirements.	
**Temporal association**				
Temporal pattern discovery (TPD)	Based on the observed‐to‐expected ratio from DP, adding a comparison of the exposed time with a control period prior to first drug exposure to identify a temporal association.^67^ Similar to LGPS.	Bayesian shrinkage to protect against spurious associations, especially for rare events.^68^ Use of chronographs for graphical interpretation, possibility to detect a greater variety of patterns.^67^	More difficult to identify patterns related to very common events^67^	
MUTARA and HUNT	MUTARA is a data‐mining algorithm looking for Unexpected Temporal Association Rules by finding any events occurring unexpectedly after the drugs of interest within a predefined risk period and excluding common events unlikely to be ADRs using a reference period to shortlist important ADRs. Treatment failure can lead to recording of events after the drug of interest, causing spurious signals. HUNT, a modified version of MUTARA, re‐ranks signals by taking account of treatment failure^69^, ^70^	Since MUTARA is event‐orientated, it should have the theoretical ability to signal infrequent ADRs^71^	MUTARA had difficulty distinguishing between adverse events and treatment failures.^72^ HUNT was developed to consider these treatment failures.	
Fuzzy‐based logic with causal‐leverage measure	This is a computation model called fuzzy recognition‐primed decision model. It uses cues such as temporal association between drugs and outcomes, rechallenenge, dechallenge to capture potential causal associations within each drug‐exposed person. The potential causalities are then incorporated into new measures (causal‐leverage and reverse causal value), which were used to rank drug‐outcome pair and remove spurious signals.^73^, ^74^	Attempt to rank the important signals by quantifying the degree of association of a drug‐outcome pair and remove background noise (spurious signals) using new measures. Developed specifically for infrequent associations.^75^	The parameter, e.g. hazard period to capture outcomes, and fuzzy variables (e.g. temporal association, rechallenge and dechallenge) could be difficult to optimise.	
**Sequence Symmetry Analysis (SSA)**	Investigate the sequence of events related to the initiation of a drug. Testing for asymmetry based on the null hypothesis that if there is no association, one would expect a symmetrical distribution of the outcome before and after the initiation of a drug.^41^	Computationally efficient, robust to confounders stable over time.^41^ Possible to evaluate all drugs and all outcomes in a database.^76^	A non‐symmetrical pattern does not necessarily indicate a signal.^41^ Triage needed to find potentially interesting associations, high number of false negatives. Assumes an appropriate single point of symmetry.	Mainly applied in large‐scale settings in the literature.^32^, ^77^, ^78^, ^79^, ^80^
**Sequential testing**: MaxSPRT, CSSP, Log Linear Model for Poisson Data (LLMP)	Suite of methods for near‐real time or prospective surveillance, building on repeated testing to detect associations. Patients are added gradually.^81^ The maxSPRT is a statistical test adjusting for multiple testing.^82^, ^83^ The CSSP method uses a conditional probability of having an outcome more extreme than the observed event rate and stratifies the population.^81^ The LLMP Is a log‐linear Poisson based method.	Ability to handle rare events.^81^ Near real‐time surveillance^83^ makes early detection of new ADRs possible, improving the timeliness of signal detection.^84^ CSSP has been developed to handle chronic exposures.^85^	The maxSPRT requires large amount of historical data to have precise estimates of the expected number of events. This problem is handled in the CSSP method.^86^ CSSP may struggle maintaining the type I error with frequent testing or numerous strata.^85^ Limited for handling continuous confounders.^81^	
**Tree‐based scan statistic**	Health events are classified in a tree hierarchical system, based on diagnoses codes at different levels. Data mining technique looks for excess risk in individual cells of the tree as well as in closely related cells.^87^ Hypothesis testing is performed using the likelihood ratio test.^88^	Simultaneous scan for signals at different levels of granularity. Minimum prior assumption about the type of health outcome of interest.^88^ Adjust for multiple hypothesis testing.^88^	Potential concern of false‐negative signals due to the control of the type I error.^89^	Has been flexibly applied with a new user, active comparator design, propensity scores techniques^64^, ^90^, ^91^ and self‐controlled designs^92^, ^93^
**Other machine learning (ML) based approaches**	Different approaches have been proposed. Some aim to train a classifier with a range of positive and negative controls.^94^ Others use data driven strategies to look for apparent outliers.^67^, ^95^	Ability to cope with large, high‐dimensional and sparse data,^96^ although computational performance can be decreased.	Need for a large training dataset. Less easily implemented by non‐specialists of ML. Relies on the ability of the learning stage to deal with noisy healthcare data.	Used in different ways for signal detection^9^, ^34^, ^36^, ^94^, ^96^, ^97^, ^98^, ^99^, ^100^, ^101^ including propensity score calculation^99^ or as a whole approach using Bradford Hill's causality considerations.^94^
**Other** Prescription Sequence Symmetry Analysis (PSSA) Methods using laboratory results	Variation of the SSA where drugs are taken as proxies for the Health Outcomes of Interest (HOIs).^32^, ^35^ Methods compared abnormal or extreme laboratory results before and after exposure to a drug.^31^			ML has also been used for prescription data only.^34^, ^36^ ML was used to combine the Comparison of Extreme Laboratory Test results (CERT), the Comparison of Extreme Abnormality Ratio (CLEAR) and the Prescription pattern Around Clinical Event (PACE) algorithms in a single value.^9^

### 3.4 Performance of the methods

Performance was defined implicitly across papers as the ability of a method to correctly detect signals among a set of positive (well‐established drug‐outcome associations) and negative controls (drugs known not to cause certain outcomes).^104^

Among the 25 performance assessment papers, 19 reported quantitative measures of performance, 6 only reported qualitative results. Such measures included the area under receiver operating the curve (AUC) in 16 of the 19 studies (84.2%), an estimate of predictive accuracy. It ranges between 0 and 1, the latter corresponding to a perfect prediction of positive controls. A value of 0.5 is identical to random guessing.^105^ The specificity, sensitivity^8^, ^36^, ^48^, ^49^, ^70^, ^81^, ^106^, ^107^ and coverage probability (proportion of the 95% confidence interval estimates that included the true parameter value, being 1 for negative controls)^48^, ^49^, ^52^, ^55^, ^60^, ^65^, ^107^, ^108^ were each used in eight papers (42.1%). The average precision^70^, ^72^, ^106^ was used in 4 studies (21.1%). The mean squared error^48^, ^49^, ^105^ and negative and positive predictive values^36^, ^106^, ^107^ were used in 3 of the 19 papers (15.8%) each, whilst the bias,^105^ partial area under the curve at 30% false‐positive rate (PAU30)^106^ and recall at 5% false‐positive rate^106^ have each been reported in two studies or less.

**FIGURE 3:**
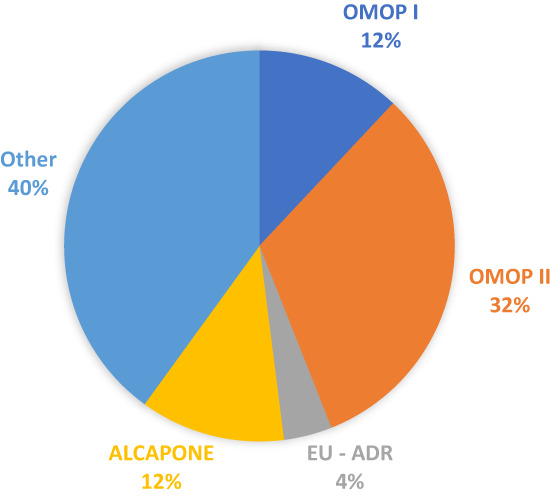
Proportion of the 25 performance assessment papers which used one of the main reference sets described in Table 4.

Fifteen of the 25 studies used datasets from three main projects, which aimed to assess the performance of methods for drug safety signal detection (Figure 3 and Table 4). Each study tested up to 126 unique parameter combinations.

**TABLE 4:** Summary of the most commonly used reference sets investigating performance of drug safety signal detection methods

Name	OMOP			
Experiment	OMOP I	OMOP II	EU‐ADR (Exploring and Understanding ADRs by integrative mining of clinical records and biomedical knowledge)	ALCAPONE (Alert generation using the case‐population approach)
**Dates**	2008–2013		2008 ‐ 2012	2016 ‐ 2018
**Country of data origin**	US		Europe (4 countries)	France
**Database(s)**	6 administrative claims (4 commercial) and 4 EHRs	4 administrative claims and 1 EHR	8 databases (EHRs)	Systeme National des Donnees de Sante (SNDS) (administrative claims)
**Size of the database(s)**	130 million	75 million	19 million	65 million
**Aims**	To test a range of methods for drug safety signal detection and determine the best strategy to implement an active drug surveillance program.		To design and assess a system to exploit EHR data for the early detection of ADRs^109^	Comparing and calibrating case‐based methods for signal detection
**Outcomes**	Angioedema, Aplastic anemia, Acute Liver injury, Bleeding, Myocardial infarction (MI), Hip fracture, Mortality after MI, Renal failure, Gastrointestinal (GI) Ulcer Hospitalization	Acute liver failure, Acute MI, Acute renal failure, Upper GI bleeding	Bullous eruptions, Acute renal failure, Anaphylactic shock, Acute MI, Rhabdomyolysis, Aplastic anaemia/pancytopenia, Neutropenia/agranulocytosis, Cardiac valve fibrosis, Acute liver injury, Upper GI bleeding	Acute Liver Failure, Acute MI, Acute Renal Failure, Upper GI bleeding
**Reference set**	53 drug–outcome pairs. 9 positive and 44 negative controls	399 drug–outcome pairs, 165 positive controls and 234 negative controls	94 drug–outcome pairs. 44 positive and 50 negative controls^22^ Same as OMOP II, 165 positive controls and 234 negative controls for the OMOP replication^110^	Combination of the OMOP II and the EU‐ADR reference set, 273 drug‐outcome pairs
**Metrics**	AUC Positive predictive value Sensitivity/Specificity Partial AUC at 30% false‐positive rate Recall at 5% false‐positive rate Average Precision	Average AUC Mean squared error “Bias” as the average difference between the log relative risk and zero for negative controls	AUC Magnitude of effect for negative controls	AUC Mean squared error Coverage probability Calibrated *p*‐values

Not all methods had their performance assessed. Disproportionality‐based methods, traditional epidemiological designs and Temporal Pattern Discovery (TPD) were evaluated in three or more papers. The tree‐based scan statistic, sequential analysis, the Mining Unexpected Temporal Association Rules (TARs) Given the Antecedent (MUTARA) and Highlighting Unexpected TARs Neglecting TARs (HUNT) algorithms were assessed in two papers or less, with few to no head‐to‐head method comparison. Some studies describing machine learning frameworks also computed measures of performance as a secondary objective, using test sets as reference standards.^9^, ^34^, ^94^, ^98^, ^101^

Seven studies presented AUC values for >1 method and a large range of drug‐outcome pairs, typically >50 pairs (Table 5). The average AUCs across all pairs and databases were as low as 0.47–0.50, below random guessing, for the New‐User Cohort and Bayesian Confidence Propagation Neural Network (BCPNN), a disproportionality‐based method. The maximum AUC was 0.81 for the Self‐Controlled Cohort. Overall, self‐controlled methods achieved higher AUCs than other methods.^4^ The High Dimensional Propensity Score (HDPS) method, used in conjunction with a new user, active comparator design achieved the highest AUCs in two papers.^66^, ^106^ TPD had higher AUCs than other methods in all studies except one,^70^ whilst MUTARA and HUNT had lower than average AUCs (0.57–0.60).^70^ The Maximized Sequential Probability Ratio Test (MaxSPRT) and the Conditional Sequential Sampling Procedure (CSSP) had low reported AUCs in the 2011 OMOP report, in the range of 0.23–0.38.^66^

**TABLE 5:** Average AUC for each method and different publications

Reference	Countries	Databases	SCCS	SCC	CC	CCO	DP	HDPS	TPD	LGPS^a^	NUC	PRR	ROR	BCPNN	GPS/MGPS
Ryan et al.^106^	United States	7 from OMOP	0.74	0.68	0.62	0.66	0.68	0.77	0.73		0.59				
Schuemie et al.^110^	Denmark, Italy, Netherlands	6 from EU‐ADR	0.67	0.75	0.61		0.6		0.67	0.59	0.59				
Ryan et al.^105^	United States	5 from OMOP	0.74	0.81	0.54		0.53		0.75	0.58	0.69				
DuMouchel et al.^52^	United States	5 from OMOP										0.57		0.50	0.54
Murphy et al.^66^	United States	PHS^2^ from OMOP	0.57		0.61	0.61	0.63	0.68	0.65		0.47				
Schuemie et al.^53^	Denmark, Italy, Netherlands	7 from EU‐ADR	0.74		0.75					0.78		0.72	0.71	0.72	0.69
Reps et al.^70^	United Kingdom	THIN							0.56				0.55		

*Note*: Where possible, the AUC is measured as the average AUC of the best performing combination across all drug‐outcome pairs and databases. Only the methods evaluated in at least two papers are displayed.

Abbreviation: PHS, partners’ healthcare system.

^a^ with LEOPARD filtering.^2^

Among Machine Learning (ML) techniques, which were not evaluated within the seven studies above, the supervised Bradford Hill had a reported AUC of 0.86,^94^ which is the highest reported average AUC among all performance assessment papers. The Longitudinal Evaluation of Observational Profiles of Adverse events Related to Drugs (LEOPARD) algorithm was found to improve the average AUCs of all methods in one study^53^ when applied to OMOP methods. A lack of differential performance between methods was observed in several papers.^53^, ^70^

One paper evaluated the performances of different algorithms for laboratory‐based signals. ML models achieved the highest AUCs (0.80–0.82), the Comparison of Extreme Laboratory Test results (CERT) and Prescription pattern Around Clinical Event (PACE) algorithms had AUCs in the range of 0.52–0.56, and disproportionality methods were the lowest performing, with AUCs of 0.52–0.56.^9^

### 3.5 Performance stratified by drug or outcome

From Table 3, no method can theoretically perform equally well for all drugs and outcomes (e.g., some methods are more suited to acute or rare outcomes). The average AUC discussed above is not representative of the full potential of a method as it represents average performance across all drug‐outcome pairs. In this section, we aim to investigate the performance of the methods for specific types of drugs and outcomes. Only 8 of the 19 quantitative performance assessment papers reported performance measures stratified by type of outcome, and one proposed an analysis per drug. One additional paper discussed stratified results qualitatively. Overall, they were not consistent in their approach.

We accessed one OMOP report which classified drugs and outcomes in subgroups,^66^ including: (1) high and low prevalence drugs and events, (2) acute and non‐acute time to event, (3) long and short exposure. The results were presented for a single database. Altogether, methods had higher AUCs with high prevalence Drugs of Interest (DOIs), except for case–control and case‐crossover. DP performed better with high prevalence DOIs than with low prevalence ones and had a high false‐positive rate with common outcomes. CSSP and MaxSPRT achieved AUCs below 0.5 for all subgroups.

Other studies provided AUC values for each of the 4 OMOP outcomes (Table 6). The Alert generation using the case‐population approach (ALCAPONE) project studied the performance of case‐based designs for upper gastrointestinal (GI) bleeding and acute liver injury. They achieved higher AUCs for acute liver injury than in OMOP. DP methods were either close or even below random guessing for different outcomes. Self‐controlled designs were consistently the best analytic choice for all databases and all outcomes in OMOP, except in one database, where TPD lead to the highest AUC for acute MI and upper GI bleed.^105^

**TABLE 6:** AUC of different methods for (a) acute liver injury, (b) acute renal failure, (c) upper gastrointestinal bleeding, and (d) acute myocardial infarction

(a) Acute liver injury						
Acute liver injury	Schuemie et al.^108^	Madigan et al.^61^	Suchard et al.^55^	Schuemie et al.^110^	DuMouchel et al.^52^	Thurin et al.^49^
Reference set	OMOP II	OMOP II	OMOP I	OMOP II	OMOP II	ALCAPONE
Country	United States	United States	United States	Europe	United States	France
LGPS + LEOPARD	0.57					
Case–control		0.59				0.90
Case‐population						0.85
SCCS			0.61	0.73		0.93
PRR					0.62	
BCPNN					0.57	
MGPS					0.50	

(b) Acute renal failure					
Acute renal failure	Schuemie et al.^108^	Madigan et al.^61^	Suchard et al.^55^	Schuemie et al.^110^	DuMouchel et al.^52^
Reference set	OMOP II	OMOP II	OMOP I	OMOP II	OMOP II
Country	United States	United States	United States	Europe	United States
LGPS + LEOPARD	0.58				
Case–control		0.62			
SCCS			0.85	0.94	
PRR					0.61
BCPNN					0.42
MGPS					0.59

(c) Upper gastrointestinal bleeding						
Upper GI bleeding	Schuemie et al.^108^	Madigan et al.^61^	Suchard et al.^55^	Schuemie et al.^110^	DuMouchel et al.^52^	Thurin et al.^48^
Reference set	OMOP II	OMOP II	OMOP I	OMOP II	OMOP II	ALCAPONE
Country	United States	United States	United States	Europe	United States	France
LGPS + LEOPARD	0.67					
Case–control		0.64				0.62
Case‐population						0.67
SCCS			0.82	0.84		0.84
PRR					0.47	
MGPS					0.53	

(d) Acute myocardial infarction					
Acute MI	Schuemie et al.^108^	Madigan et al.^61^	Suchard et al.^55^	Schuemie et al.^110^	DuMouchel et al.^52^
Reference set	OMOP II	OMOP II	OMOP I	OMOP II	OMOP II
Country	United States	United States	United States	Europe	United States
LGPS + LEOPARD	0.662				
Case–control		0.65			
SCCS			0.73	0.79	
PRR					0.60
MGPS					0.60

Abbreviation: Gastrointestinal (GI); LEOPARD, Longitudinal Evaluation of Observational Profiles of Adverse events Related to Drugs; LGPS, Longitudinal Gamma Poisson Shrinker; MGPS, Multi‐Item Gamma Poisson Shrinker; PRR, Proportional Reporting Ratio; Self‐controlled case series (SCCS).

In Zhou et al.,^56^ the Self‐Controlled Case Series (SCCS) was able to highlight all acute events of interest in the primary analysis, such as fractures or GI perforation, including some outcomes that were not explored in other projects. Regarding slower onset outcomes, two were not highlighted but the association between adalimumab and lymphoma was signaled.

Several studies explored slower onset outcomes including cancer using a case–control design^111^, ^112^, ^113^, ^114^ and one paper used a case‐crossover design^115^ but none reported performance. Kulldorff et al.^88^ mentioned the possibility to use the tree‐based scan statistic with chronic events but this has not been tested so far.

According to several studies,^70^, ^72^ many methods achieved low performances with rare ADRs. A study^72^ found that all MUTARA, HUNT, and reporting odds ratio (ROR) did not achieve a higher mean average precision (MAP) than 0.03 when restricted to rare ADRs, compared to MAPs ranging from 0.04 to 0.09 for all outcomes.

Only one performance assessment paper took a drug‐based approach, investigating 6 drug families with various lengths of treatment (short vs. long term). They computed TPD, HUNT, MUTARA and ROR. However, no differential pattern of performance was observed.^70^

## 4 DISCUSSION

### 4.1 Principal findings

There is an increasing interest in implementing RWD in signal detection (Figure 2) and several major initiatives have contributed to advances in methods development and performance assessment. However, performance assessment was heterogeneous, with a lack of agreement on the definition of a gold standard and what good performance looks like, making comparison difficult across methods, studies and data sources.

### 4.2 Overall performance

Overall, the self‐controlled methods tended to achieve higher AUCs than other methods, including case–control and disproportionality ones. The results were consistent across several OMOP papers and their replication in Europe. The HDPS and TPD methods also achieved higher AUCs, both on average and in certain subgroups. However, they were not evaluated in many studies and their running time was longer than for other methods.^66^ Disproportionality methods, widely used in SRs, seem not to be able to distinguish between positive and negative controls as they had reported AUCs close to random guessing.^52^ This result was anticipated as SRs have different properties to that of RWD. Although the tree‐based scan statistic did not undergo a formal performance assessment, it was able to capture known signals^88^ and could be useful for assessing outcomes at different levels of granularity, particularly in a drug‐based approach. Similarly, performance of ML has been evaluated heterogeneously, but preliminary results highlight its potential for signal detection.

Performance measures were generally reported on average across all drugs and outcomes in the reference set, even though every epidemiological study design performs better with some exposure and outcome types than others. Therefore, reported overall performance could hide particularly strong or weak performance for sets of similar exposure–outcomes combinations.

Performance was mainly assessed and presented with the AUC, which is a single measure and does not incorporate aspects such as bias.^116^ It assumes that every threshold of sensitivity and specificity is equally important, which in practice is not the case for signal detection, and while objective in practice may provide a misleading view of signal detection value. Other measures were sparsely reported and could not be compared.

### 4.3 Performance stratified by type of drug or outcome

Only 10 papers proposed an analysis by subgroup of drugs and outcomes, in a heterogeneous manner. It is encouraging to see increased performances in subgroup analyses compared to the average AUCs reported earlier, meaning that some methods are able to perform well when restricted to certain subgroups of DOIs and HOIs. Further work is needed to assess the reliability and reproducibility of these results.

Self‐controlled methods were optimal for all acute outcomes in OMOP^105^ expect in one of the databases where TPD led to the highest AUCs. Zhou et al.^56^ supported these results and suggested that self‐controlled methods may identify slow onset outcomes if the signal is strong. However, they did not investigate negative controls so the specificity of their findings is unknown.

Most of the papers were non‐specific in their selection of outcome and its characterization or focused on rapid onset AEs. The best method for detecting long‐term ADRs, if any to date, remains understudied and therefore unclear. Further work is needed in this area as routinely collected data can have a great advantage of recording long‐term outcomes over SRs. Since they can happen years after exposure, it is clearly an even more difficult signal detection problem to associate the outcome with a drug exposure with SRs.

### 4.4 Comparability and generalizability of the findings

There was a lack of agreement on a possible gold standard for performance assessment. The findings were strongly influenced by the three main projects described earlier since most of the studies used one of the specific references sets that were proposed therein, which while large still represent a small proportion of all safety knowledge and have well published limitations.^23^, ^117^, ^118^ These reference sets used different outcome definitions. Some were limited to strong signals, and slower onset outcomes were mostly excluded.

There is an inherent variation of the AUCs between the databases, which was shown to be 20‐30% for each method between U.S. databases in the OMOP experiment with the same reference set.^106^ Comparison across studies using different databases is therefore not possible. However, study replication in several databases can increase precision and power to detect certain signals.^119^

Signal detection capabilities also depend greatly on the chosen analytic configuration.^4^, ^24^, ^105^ In Ryan et al.,^105^ at least one configuration led to an AUC close or equal to 0.5 for each method‐drug‐outcome combination. In this review, the optimal configuration across all outcomes and databases was chosen as the reference measure, but higher AUCs could be achieved when applying the optimal configuration to a single outcome and database. Gruber et al.^23^ suggested that design choices need to be specific to the characteristics of the drug outcome pairs to avoid highlighting spurious associations.

### 4.5 Strengths and limitations of the review

To our knowledge, this is the first systematic review to explore the performance of methods for signal detection stratified by drugs and outcomes. Moreover, we updated the literature by including methods that are recently developed. We comprehensively described methods used for signal detection, evaluated the quality of the included studies narratively as well as compared the main measures of performance reported from the literature.

We also recognize some limitations. First, relevant studies might have been missed if they did not mention specific keywords in their abstract or full text as signal detection terminology is not standardly used in current literature. We added manual searching and screening bibliography of reviews to improve sensitivity. Quantitative comparison of performance was limited by the heterogeneity of the publications and the lack of gold standard, replicability of the studies was insufficient to perform re‐analyses.

### 4.6 Recommendations

Further research on the methods' performances for specific types of drugs and outcomes, focusing on inherent strengths and limitations of each method is needed. We also encourage more comprehensive reporting of the performance for individual or subgroups of drug–outcome pairs. We would like to see more head to head comparisons of methods for a larger range of drug–outcome pairs, including slower‐onset outcomes. As all reference sets have inherent limitations, we would encourage the development of multiple and diverse reference sets publicly available for reuse. Ideally, generic and accessible codes that can be implemented in any database could be developed, with the use of common data models. We would also like to see results on the timeliness of signal detection with RWD, which was investigated only a single paper included in this review.^15^

## 5 CONCLUSIONS

No method using routinely collected data showed superior performance across all drugs and outcomes, with heterogeneous performance assessment and reporting. However, some evidence showed that self‐controlled designs, HDPS and ML achieved higher AUCs compared to other methods. Performance assessment for methods with slower onset outcomes is lacking.

An ideal approach is likely to involve more than one method to detect multiple drug–outcome pairs since none appears to have universal application to all outcomes and drugs. The aim of a signal detection programme, the type of drugs and outcomes under consideration and the drug‐ or outcome‐based approach taken should be guiding the choice of the method. Future studies should investigate the performance of methods stratified by type of drug and outcome.

## CONFLICT OF INTEREST

Astrid Coste is funded by a GSK PhD studentship to undertake this review. Andrew Bate is an employee of GSK and holds stocks and stock options. Ian Douglas holds grants and shares from GSK.

## Supporting information


**Supplementary S1** ‐ List of included original studies and reviews

**Appendix S1** – Search strategies in the different databases
