# Supplementary material for: Methods for drug safety signal detection using routinely collected observational electronic health care data: A systematic review
Source: Pharmacoepidemiol Drug Saf. 2022 Nov 2;32(1):28–43. doi: 10.1002/pds.5548 (PMC10092128; doi:10.1002/pds.5548)
Supplement: Supplementary file 1 — Supplementary S1 ‐ List of included original studies and reviews [file PDS-32-28-s001.docx]

**Supplement 1 - List of included original studies and reviews**

1. Moore TJ, Furberg CD. Electronic Health Data for Postmarket Surveillance: A Vision Not Realized. *Drug Saf* 2015; **38**: 601–610. doi:https://dx.doi.org/10.1007/s40264-015-0305-9.

2. Kulldorff M, Wang S v, Vine S, *et al.* A novel data mining application to detect safety signals for newly approved medications in routine care of patients with diabetes. *Endocrinology, Diabetes and Metabolism* 2021. doi:http://dx.doi.org/10.1002/edm2.237.

3. Escolano S, Tubert-Bitter P, Ahmed I, Demailly R, Haramburu F. Identifying Drugs Inducing Prematurity by Mining Claims Data with High-Dimensional Confounder Score Strategies. *Drug Safety* 2020; **43**: 549–559. doi:http://dx.doi.org/10.1007/s40264-020-00916-5.

4. Ferrajolo C, Verhamme KMC, Trifirò G, *et al.* Idiopathic acute liver injury in paediatric outpatients: Incidence and signal detection in two european countries. *Drug Safety* 2013; **36**: 1007–1016. doi:http://dx.doi.org/10.1007/s40264-013-0045-7.

5. Ryan PB, Stang PE, Overhage JM, *et al.* A comparison of the empirical performance of methods for a risk identification system. *Drug Saf* 2013; **36 Suppl 1**: S143-58. doi:https://dx.doi.org/10.1007/s40264-013-0108-9.

6. Ji Y, Ying H, Dews P, *et al.* An exclusive causal-leverage measure for detecting adverse drug reactions from electronic medical records. In: *Annual Conference of the North American Fuzzy Information Processing Society - NAFIPS*., 2011. doi:10.1109/NAFIPS.2011.5751957.

7. Islam T, Hussain N, Islam S, Chakrabarty A. Detecting adverse drug reaction with data mining and predicting its severity with machine learning. In: *IEEE Region 10 Humanitarian Technology Conference, R10-HTC*.Vol 2018-Decem., 2019. doi:10.1109/R10-HTC.2018.8629806.

8. Jin H, Chen J, Kelman C, He H, McAullay D, O’Keefe CM. Mining unexpected associations for signalling potential adverse drug reactions from administrative health databases. *Lecture Notes in Computer Science (including subseries Lecture Notes in Artificial Intelligence and Lecture Notes in Bioinformatics)* 2006; **3918 LNAI**: 867–876. doi:10.1007/11731139_101.

9. Reps J, Feyereisl J, Garibaldi JM, Aickelin U, Gibson JE, Hubbard RB. Investigating the detection of adverse drug events in a UK general practice electronic health-care database. In: *UKCI 2011 - Proceedings of the 11th UK Workshop on Computational Intelligence*., 2011; 167–172. Available at: https://www.scopus.com/inward/record.uri?eid=2-s2.0-84908489832&partnerID=40&md5=95653c8a28085895d4585dc0bc200c7f.

10. Lo HZ, Ding W, Nazeri Z. Mining adverse drug reactions from electronic health records. In: *Proceedings - IEEE 13th International Conference on Data Mining Workshops, ICDMW 2013*., 2013; 1137–1140. doi:10.1109/ICDMW.2013.43.

11. Kuang Z, Peissig P, Costa VS, Maclin R, Page D. Pharmacovigilance via baseline regularization with large-scale longitudinal observational data. In: *Proceedings of the ACM SIGKDD International Conference on Knowledge Discovery and Data Mining*.Vol Part F1296., 2017; 1537–1546. doi:10.1145/3097983.3097998.

12. Reps J, Garibaldi JM, Aickelin U, Soria D, Gibson JE, Hubbard RB. Comparing data-mining algorithms developed for longitudinal observational databases. In: *2012 12th UK Workshop on Computational Intelligence, UKCI 2012*., 2012. doi:10.1109/UKCI.2012.6335771.

13. Bampa M, Papapetrou P. Mining adverse drug events using multiple feature hierarchies and patient history windows. In: *IEEE International Conference on Data Mining Workshops, ICDMW*.Vol 2019-Novem., 2019; 925–932. doi:10.1109/ICDMW.2019.00135.

14. Karlsson I, Zhao J. Dimensionality reduction with random indexing: An application on adverse drug event detection using electronic health records. In: *Proceedings - IEEE Symposium on Computer-Based Medical Systems*., 2014; 304–307. doi:10.1109/CBMS.2014.22.

15. Mansour A, Ying H, Dews P, Ji Y, Michael Massanari R. Fuzzy rule-based approach for detecting adverse drug reaction signal pairs. In: *8th Conference of the European Society for Fuzzy Logic and Technology, EUSFLAT 2013 - Advances in Intelligent Systems Research*.Vol 32., 2013; 384–391. Available at: https://www.scopus.com/inward/record.uri?eid=2-s2.0-84891771416&partnerID=40&md5=78ef71b30f239c3d773639c5fd4cfcab.

16. Reps JM, Garibaldi JM, Aickelin U, Soria D, Gibson J, Hubbard R. Comparison of algorithms that detect drug side effects using electronic healthcare databases. *Soft Computing* 2013; **17**: 2381–2397. doi:10.1007/s00500-013-1097-4.

17. Karimi S, Wang C, Metke-Jimenez A, Gaire R, Paris C. Text and data mining techniques in adverse drug reaction detection. *ACM Computing Surveys* 2015; **47**. doi:10.1145/2719920.

18. Lo H, Ding W, Nazeri Z. Temporality and Context for detecting adverse drug reactions from longitudinal data. *Applied Intelligence* 2014; **41**: 1069–1080. doi:10.1007/s10489-014-0568-3.

19. Zhan C, Liu L, Li J, Roughead E, Pratt N. Detecting potential signals of adverse drug events from prescription data. *Artificial Intelligence in Medicine* 2020; **104**: 101839. doi:http://dx.doi.org/10.1016/j.artmed.2020.101839.

20. Kaguelidou F, Durrieu G, Clavenna A. Pharmacoepidemiological research for the development and evaluation of drugs in pediatrics. *Therapie* 2019; **74**: 315–324. doi:http://dx.doi.org/10.1016/j.therap.2018.09.077.

21. Maric I, Weber KA, Wong RJ, *et al.* Data-driven queries between medications and spontaneous preterm birth among 2.5 million pregnancies. *Birth Defects Research* 2019; **111**: 1145–1153. doi:http://dx.doi.org/10.1002/bdr2.1580.

22. Coloma PM, Verhamme KMC, Schuemie MJ, *et al.* Signal detection of potentially drug-induced acute liver injury in children using a multi-country healthcare database network. *Drug Safety* 2014; **37**: 99–108. doi:http://dx.doi.org/10.1007/s40264-013-0132-9.

23. Jones JK. The role of data mining technology in the identification of signals of possible adverse drug reactions: Value and limitations. *Current Therapeutic Research - Clinical and Experimental* 2001; **62**: 664–672. doi:http://dx.doi.org/10.1016/S0011-393X%2801%2980072-2.

24. Friedman GD, Ury HK. Screening for possible drug carcinogenicity: Second report of findings. *J Natl Cancer Inst* 1983; **71**: 1165–1175. Available at: http://ovidsp.ovid.com/ovidweb.cgi?T=JS&PAGE=reference&D=emed3&NEWS=N&AN=14182047.

25. Thurin NH, Lassalle R, Schuemie M, *et al.* Empirical assessment of case-based methods for drug safety alert identification in the French National Healthcare System database (SNDS): Methodology of the ALCAPONE project. *Pharmacoepidemiol Drug Saf* 2020; **29**: 993–1000. doi:https://dx.doi.org/10.1002/pds.4983.

26. Reich CG, Ryan PB, Schuemie MJ. Alternative outcome definitions and their effect on the performance of methods for observational outcome studies. *Drug Saf* 2013; **36 Suppl 1**: S181-93. doi:https://dx.doi.org/10.1007/s40264-013-0111-1.

27. Li L. A conditional sequential sampling procedure for drug safety surveillance. *Statistics in Medicine* 2009; **28**: 3124–3138. doi:10.1002/sim.3689.

28. Wei R, Jia LL, Yu YC, *et al.* Pediatric drug safety signal detection of non-chemotherapy drug-induced neutropenia and agranulocytosis using electronic healthcare records. *Expert Opinion on Drug Safety* 2019; **18**: 435–441. doi:10.1080/14740338.2019.1604682.

29. Yu Y-C, Wei R, Jia L-L, *et al.* Exploring the drug-induced anemia signals in children using electronic medical records. *Expert Opinion on Drug Safety* 2019; **18**: 993–999. doi:http://dx.doi.org/10.1080/14740338.2019.1645832.

30. Choi N-K, Chang Y, Choi YK, Hahn S, Park B-J. Signal detection of rosuvastatin compared to other statins: data-mining study using national health insurance claims database. *Pharmacoepidemiology and Drug Safety* 2010; **19**: 238–246. Available at: http://www3.interscience.wiley.com/cgi-bin/fulltext/123229204/PDFSTART.

31. Madigan D, Schuemie MJ, Ryan PB. Empirical performance of the case-control method: Lessons for developing a risk identification and analysis system. *Drug Safety* 2013; **36**: S73–S82. Available at: http://ovidsp.ovid.com/ovidweb.cgi?T=JS&PAGE=reference&D=emed14&NEWS=N&AN=372188894. Accessed July 13, 2021.

32. Schuemie MJ, Madigan D, Ryan PB. Empirical performance of LGPS and LEOPARD: Lessons for developing a risk identification and analysis system. *Drug Safety* 2013; **36**: S133–S142. Available at: http://ovidsp.ovid.com/ovidweb.cgi?T=JS&PAGE=reference&D=emed14&NEWS=N&AN=372188899. Accessed July 13, 2021.

33. Ryan PB, Schuemie MJ, Gruber S, Zorych I, Madigan D. Empirical performance of a new user cohort method: Lessons for developing a risk identification and analysis system. *Drug Safety* 2013; **36**: S59–S72. Available at: http://ovidsp.ovid.com/ovidweb.cgi?T=JS&PAGE=reference&D=emed14&NEWS=N&AN=372188893. Accessed July 13, 2021.

34. Pacurariu AC, Straus SM, Trifirò G, *et al.* Useful Interplay Between Spontaneous ADR Reports and Electronic Healthcare Records in Signal Detection. *Drug Safety* 2015; **38**: 1201–1210. Available at: http://rd.springer.com/journal/40264.

35. Wang S v., Gagne JJ, Schneeweiss S, *et al.* Hypothesis-free screening of large administrative databases for unsuspected drug-outcome associations. *European Journal of Epidemiology* 2018; **33**: 545–555. Available at: http://www.wkap.nl/journalhome.htm/0393-2990. Accessed July 5, 2021.

36. Nelson JC, Ulloa-Pèrez E, Bobb JF, *et al.* Leveraging the entire cohort in drug safety monitoring: part 1 methods for sequential surveillance that use regression adjustment or weighting to control confounding in a multisite, rare event, distributed data setting. *Journal of Clinical Epidemiology* 2019; **112**: 77–86. Available at: http://www.elsevier.com/locate/jclinepi. Accessed July 13, 2021.

37. Schuemie MJ, Trifirò G, Coloma PM, Ryan PB, Madigan D. Detecting adverse drug reactions following long-term exposure in longitudinal observational data: The exposure-adjusted self-controlled case series. *Statistical Methods in Medical Research* 2016; **25**: 2577–2592. Available at: http://smm.sagepub.com/archive/. Accessed July 13, 2021.

38. Tham MY, Ye Q, Ang PS, *et al.* Application and optimisation of the Comparison on Extreme Laboratory Tests (CERT) algorithm for detection of adverse drug reactions: Transferability across national boundaries. *Pharmacoepidemiology and Drug Safety* 2018; **27**: 87–94. Available at: http://onlinelibrary.wiley.com/journal/10.1002/(ISSN)1099-1557. Accessed July 13, 2021.

39. Wang S v, Gagne JJ, Patorno E, *et al.* A General Propensity Score for Signal Identification Using Tree-Based Scan Statistics. *American Journal of Epidemiology* 2021; **190**: 1424–1433. Available at: http://www.ncbi.nlm.nih.gov/pubmed/33615330. Accessed July 13, 2021.

40. Coloma PM, Trifirò G, Schuemie MJ, *et al.* Electronic healthcare databases for active drug safety surveillance: is there enough leverage? 2012; **21**: 611–621. Available at: http://ovidsp.ovid.com/ovidweb.cgi?T=JS&PAGE=reference&D=med9&NEWS=N&AN=22315152. Accessed July 5, 2021.

41. Wahab IA, Pratt NL, Kalisch LM, Roughead EE. Comparing time to adverse drug reaction signals in a spontaneous reporting database and a claims database: a case study of rofecoxib-induced myocardial infarction and rosiglitazone-induced heart failure signals in Australia. *Drug Safety* 2014; **37**: 53–64. Available at: http://ovidsp.ovid.com/ovidweb.cgi?T=JS&PAGE=reference&D=emed15&NEWS=N&AN=372515382.

42. Coloma PM, Schuemie MJ, van Mulligen E, *et al.* Drug-Induced Acute Myocardial Infarction: Identifying ‘Prime Suspects’ from Electronic Healthcare Records- Based Surveillance System. *Plos One* 2013; **8**: e72148. Available at: http://ovidsp.ovid.com/ovidweb.cgi?T=JS&PAGE=reference&D=med10&NEWS=N&AN=24015213. Accessed July 13, 2021.

43. Schuemie MJ, Coloma PM, Straatman H, *et al.* Using Electronic Health Care Records for Drug Safety Signal Detection: A Comparative Evaluation of Statistical Methods. *Medical Care* 2012; **50**: 890–897. doi:10.1097/MLR.0B013E31825F63BF.

44. Park MY, Yoon D, Lee K, *et al.* A novel algorithm for detection of adverse drug reaction signals using a hospital electronic medical record database. *Pharmacoepidemiology and Drug Safety* 2011; **20**: 598–607. Available at: http://ovidsp.ovid.com/ovidweb.cgi?T=JS&PAGE=reference&D=med8&NEWS=N&AN=21472818. Accessed July 13, 2021.

45. Duan L, Khoshneshin M, Street WN, Liu M. Adverse drug effect detection. *IEEE Journal of Biomedical and Health Informatics* 2013; **17**: 305–311. Available at: http://ovidsp.ovid.com/ovidweb.cgi?T=JS&PAGE=reference&D=med10&NEWS=N&AN=24235108. Accessed July 5, 2021.

46. Norén N, Hopstadius J, Bate A, Edwards IR. Safety surveillance of longitudinal databases: Methodological considerations. *Pharmacoepidemiology and Drug Safety* 2011; **20**: 714–717. doi:10.1002/pds.2151.

47. Brown JS, Kulldorff M, Petronis KR, *et al.* Early adverse drug event signal detection within population-based health networks using sequential methods: Key methodologic considerations. *Pharmacoepidemiology and Drug Safety* 2009; **18**: 226–234. Available at: http://www3.interscience.wiley.com/cgi-bin/fulltext/121638545/PDFSTART. Accessed July 5, 2021.

48. Patadia VK, Schuemie MJ, Coloma P, *et al.* Evaluating performance of electronic healthcare records and spontaneous reporting data in drug safety signal detection. *International Journal of Clinical Pharmacy* 2015; **37**: 94–104. Available at: http://springerlink.metapress.com/content/2210-7703/.

49. Morel M, Bacry E, Gaïffas S, *et al.* ConvSCCS: convolutional self-controlled case series model for lagged adverse event detection. *Biostatistics* 2020; **21**: 758–774. Available at: http://ovidsp.ovid.com/ovidweb.cgi?T=JS&PAGE=reference&D=emexa&NEWS=N&AN=633182054. Accessed July 13, 2021.

50. Lee S, Choi J, Kim H-SS, *et al.* Standard-based comprehensive detection of adverse drug reaction signals from nursing statements and laboratory results in electronic health records. *Journal of the American Medical Informatics Association* 2017; **24**: 697–708. doi:10.1093/JAMIA/OCW168.

51. Pratt N, Chan EW, Choi N-KK, *et al.* Prescription sequence symmetry analysis: Assessing risk, temporality, and consistency for adverse drug reactions across datasets in five countries. *Pharmacoepidemiology and Drug Safety* 2015; **24**: 858–864. Available at: http://onlinelibrary.wiley.com/journal/10.1002/(ISSN)1099-1557. Accessed July 13, 2021.

52. Wintzell V, Svanström H, Melbye M, *et al.* Data Mining for Adverse Events of Tumor Necrosis Factor-Alpha Inhibitors in Pediatric Patients: Tree-Based Scan Statistic Analyses of Danish Nationwide Health Data. *Clinical Drug Investigation* 2020; **40**: 1147–1154. Available at: http://rd.springer.com/journal/40261. Accessed July 13, 2021.

53. Pottegård A, Friis S, dePont Christensen R, Habel LA, Gagne JJ, Hallas J. Identification of Associations Between Prescribed Medications and Cancer: A Nationwide Screening Study. *EBioMedicine* 2016; **7**: 73–79. doi:10.1016/j.ebiom.2016.03.018.

54. Whalen E, Hauben M, Bate Andrew. Time Series Disturbance Detection for Hypothesis-Free Signal Detection in Longitudinal Observational Databases. San-Jose A Vidal X, Aguilera C, Ballarin E, Perez E, Barbe J, Bocanegra CP, Toscano A, Pal C, Teixidor T, Fernandez-Moyano A, Hernandez MG, de la Rosa Morales R, Martinez MNB, Lopez-Soto A, Bosch X, Palau MJ, Rovira J, Navarro M, Formiga F, Chivite D, Ro AA (ed.). *Drug Saf* 2018; **22**: 890–897. doi:10.1097/MLR.0b013e31825f63bf.

55. Norén GN, Hopstadius J, Bate A, Star K, Edwards IR. Temporal pattern discovery in longitudinal electronic patient records. *DATA MINING AND KNOWLEDGE DISCOVERY* 2010; **20**: 361–387. doi:10.1007/s10618-009-0152-3.

56. Patadia VK, Coloma P, Schuemie MJ, *et al.* Using real-world healthcare data for pharmacovigilance signal detection-the experience of the EU-ADR project. *Expert Review of Clinical Pharmacology* 2015; **8**: 95–102. doi:http://dx.doi.org/10.1586/17512433.2015.992878.

57. Zhou X, Douglas IanJ, Shen Rongjun, Bate Andrew, Douglas IanJ, Bate Andrew. Signal Detection for Recently Approved Products: Adapting and Evaluating Self-Controlled Case Series Method Using a US Claims and UK Electronic Medical Records Database. *Drug Safety* 2018; **41**: 523–536. Available at: http://rd.springer.com/journal/40264. Accessed July 13, 2021.

58. Schachterle SE, Hurley S, Liu Q, Petronis KR, Bate A. An Implementation and Visualization of the Tree-Based Scan Statistic for Safety Event Monitoring in Longitudinal Electronic Health Data. *Drug Safety* 2019; **42**: 727–741. doi:http://dx.doi.org/10.1007/s40264-018-00784-0.

59. Norén GN, Bergvall T, Ryan PB, *et al.* Empirical performance of the calibrated self-controlled cohort analysis within temporal pattern discovery: Lessons for developing a risk identification and analysis system. *Drug Safety* 2013; **36**: S107–S121. doi:10.1007/s40264-013-0095-x.

60. Lai EC-C, Pratt N, Hsieh C-Y, *et al.* Sequence symmetry analysis in pharmacovigilance and pharmacoepidemiologic studies. *European Journal of Epidemiology* 2017; **32**: 567–582. doi:http://dx.doi.org/10.1007/s10654-017-0281-8.

61. Zhan C, Liu L, Li J, Roughead E, Pratt N. A data-driven method to detect adverse drug events from prescription data. *Journal of Biomedical Informatics* 2018; **85**: 10–20. Available at: http://www.elsevier.com/inca/publications/store/6/2/2/8/5/7/index.htt. Accessed July 5, 2021.

62. Ryan PB, Madigan D, Stang PE, *et al.* Empirical assessment of methods for risk identification in healthcare data: results from the experiments of the Observational Medical Outcomes Partnership. *Stat Med* 2012; **31**: 4401–4415. doi:https://dx.doi.org/10.1002/sim.5620.

63. Schuemie MJ, Gini R, Coloma PM, *et al.* Replication of the OMOP experiment in europe: Evaluating methods for risk identification in electronic health record databases. *Drug Safety* 2013; **36**: 159–169. doi:10.1007/s40264-013-0109-8.

64. Wahab IA, Pratt NL, Kalisch Ellett L, Roughead EE. Sequence Symmetry Analysis as a Signal Detection Tool for Potential Heart Failure Adverse Events in an Administrative Claims Database. *Drug Safety* 2016; **39**: 347–354. doi:10.1007/s40264-015-0391-8.

65. Sauer B, Nebeker J, Shen S, *et al.* Methodological framework to identify possible adverse drug reactions using population-based administrative data. *F1000Res* 2014; **3**: 258. doi:10.12688/f1000research.4816.1.

66. Zorych I, Madigan D, Ryan P, Bate A. Disproportionality methods for pharmacovigilance in longitudinal observational databases. *Statistical Methods in Medical Research* 2013; **22**: 39–56. doi:10.1177/0962280211403602.

67. Zhou X, Bao W, Gaffney M, Shen R, Young S, Bate A. Assessing performance of sequential analysis methods for active drug safety surveillance using observational data. *Journal of Biopharmaceutical Statistics* 2018; **28**: 668–681. Available at: http://www.tandf.co.uk/journals/titles/10543406.asp. Accessed July 5, 2021.

68. Coloma PM, Trifirò G, Patadia V, Sturkenboom M. *Postmarketing safety surveillance: Where does signal detection using electronic healthcare records fit into the big picture?*, 2013; 183–197.

69. Arnaud M, Bégaud B, Thiessard F, *et al.* An Automated System Combining Safety Signal Detection and Prioritization from Healthcare Databases: A Pilot Study. *Drug Safety* 2018; **41**: 377–387.

70. Curtis JR, Cheng H, Delzell E, *et al.* Adaptation of bayesian data mining algorithms to longitudinal claims data: Coxib safety as an example. *Medical Care* 2008; **46**: 969–975.

71. Brown JS, Kulldorff M, Chan KA, *et al.* Early detection of adverse drug events within population-based health networks: Application of sequential testing methods. *Pharmacoepidemiology and Drug Safety* 2007; **16**: 1275–1284.

72. Coloma PM, Avillach P, Salvo F, *et al.* A reference standard for evaluation of methods for drug safety signal detection using electronic healthcare record databases. *Drug Saf* 2013; **36**: 13–23. doi:10.1007/s40264-012-0002-x.

73. Arnaud M, Bégaud B, Thurin N, Moore N, Pariente A, Salvo F. *Methods for safety signal detection in healthcare databases: a literature review*. Taylor and Francis Ltd, 2017; 721–732. doi:10.1080/14740338.2017.1325463.

74. Hoang T, Liu J, Roughead E, Pratt N, Li J. Supervised signal detection for adverse drug reactions in medication dispensing data. *Computer Methods and Programs in Biomedicine* 2018; **161**: 25–38.

75. Ji Y, Ying H, Dews P, *et al.* A potential causal association mining algorithm for screening adverse drug reactions in postmarketing surveillance. *IEEE Trans Inf Technol Biomed* 2011; **15**: 428–437. doi:http://dx.doi.org/10.1109/TITB.2011.2131669.

76. Wisniewski AFZZ, Bate A, Bousquet C, *et al.* Good Signal Detection Practices: Evidence from IMI PROTECT. 2016; **39**: 469–490. Available at: http://ovidsp.ovid.com/ovidweb.cgi?T=JS&PAGE=reference&D=med13&NEWS=N&AN=26951233. Accessed July 13, 2021.

77. Suchard MA, Zorych I, Simpson SE, Madigan D, Schuemie MJ, Ryan PB. Empirical performance of the self-controlled case series design: Lessons for developing a risk identification and analysis system. *Drug Safety* 2013; **36**: S83–S93. Available at: http://ovidsp.ovid.com/ovidweb.cgi?T=JS&PAGE=reference&D=emed14&NEWS=N&AN=372188895. Accessed July 13, 2021.

78. Zhao J, Henriksson A, Kvist M, Asker L, Boström H. Handling Temporality of Clinical Events for Drug Safety Surveillance. *AMIA  Annual Symposium proceedings* 2015; **2015**: 1371–1380. Available at: http://ovidsp.ovid.com/ovidweb.cgi?T=JS&PAGE=reference&D=med12&NEWS=N&AN=26958278.

79. Reps JM, Garibaldi JM, Aickelin U, Gibson JE, Hubbard RB. A supervised adverse drug reaction signalling framework imitating Bradford Hill’s causality considerations. *Journal of Biomedical Informatics* 2015; **56**: 356–368. doi:http://dx.doi.org/10.1016/j.jbi.2015.06.011.

80. Bagattini F, Karlsson I, Rebane J, Papapetrou P. A classification framework for exploiting sparse multi-variate temporal features with application to adverse drug event detection in medical records. *BMC Medical Informatics and Decision Making* 2019; **19**.

81. Sauzet O, Carvajal A, Escudero A, Molokhia M, Cornelius VR. Illustration of the weibull shape parameter signal detection tool using electronic healthcare record data. *Drug Safety* 2013; **36**: 995–1006. Available at: http://ovidsp.ovid.com/ovidweb.cgi?T=JS&PAGE=reference&D=emed14&NEWS=N&AN=370010174. Accessed July 13, 2021.

82. Schuemie MJ. Methods for drug safety signal detection in longitudinal observational databases: LGPS and LEOPARD. *Pharmacoepidemiology and Drug Safety* 2011; **20**: 292–299. Available at: http://ovidsp.ovid.com/ovidweb.cgi?T=JS&PAGE=reference&D=emed12&NEWS=N&AN=361328218. Accessed July 13, 2021.

83. Brown JS, Petronis KR, Bate A, *et al.* Drug Adverse Event Detection in Health Plan Data Using the Gamma Poisson Shrinker and Comparison to the Tree-based Scan Statistic. *Pharmaceutics* 2013; **5**: 179–200. doi:10.3390/pharmaceutics5010179.

84. Hellfritzsch M, Rasmussen L, Hallas J, Pottegard A. Using the Symmetry Analysis Design to Screen for Adverse Effects of Non-vitamin K Antagonist Oral Anticoagulants. *Drug Safety* 2018; **41**: 685–695. doi:http://dx.doi.org/10.1007/s40264-018-0650-6.

85. Choi N-K, Chang Y, Kim J-Y, Choi Y-K, Park B-J. Comparison and validation of data-mining indices for signal detection: Using the Korean national health insurance claims database. *Pharmacoepidemiology and Drug Safety* 2011; **20**: 1278–1286. doi:http://dx.doi.org/10.1002/pds.2237.

86. DuMouchel W, Ryan PB, Schuemie MJ, Madigan D. Evaluation of disproportionality safety signaling applied to healthcare databases. *Drug Safety* 2013; **36**: S123–S132. Available at: http://ovidsp.ovid.com/ovidweb.cgi?T=JS&PAGE=reference&D=emed14&NEWS=N&AN=372188898. Accessed July 13, 2021.

87. Prieto-Merino D, Quartey G, Wang J, Kim J. Why a Bayesian approach to safety analysis in pharmacovigilance is important. *Pharmaceutical Statistics* 2011; **10**: 554–559. Available at: http://ovidsp.ovid.com/ovidweb.cgi?T=JS&PAGE=reference&D=emed12&NEWS=N&AN=51755107. Accessed July 13, 2021.

88. Suling M, Pigeot I. Signal Detection and Monitoring Based on Longitudinal Healthcare Data. *Pharmaceutics* 2012; **4**: 607–640. Available at: http://www.mdpi.com/1999-4923/4/4/607/pdf. Accessed July 13, 2021.

89. Jeong E, Park N, Choi Y, Park RW, Yoon D. Machine learning model combining features from algorithms with different analytical methodologies to detect laboratory-event-related adverse drug reaction signals. *PLoS ONE* 2018; **13**: e0207749. Available at: https://journals.plos.org/plosone/article/file?id=10.1371/journal.pone.0207749&type=printable. Accessed July 13, 2021.

90. Kulldorff M, Dashevsky I, Avery T, *et al.* Drug safety data mining with a tree-based scan statistic. *Pharmacoepidemiology and Drug Safety* 2013; **19**: S172–S173. doi:http://dx.doi.org/10.1002/pds.2019.

91. Hopstadius J, Noren GN, Bate A. Shrinkage observed-to-expected ratios for robust and transparent large-scale pattern discovery. *Statistical Methods in Medical Research* 2013; **22**: 57–69. doi:10.1177/0962280211403604.

92. Kim J, Kim MMJ, Ha J-H, *et al.* Signal detection of methylphenidate by comparing a spontaneous reporting database with a claims database. *Regulatory Toxicology and Pharmacology* 2011; **61**: 154–160. doi:http://dx.doi.org/10.1016/j.yrtph.2011.03.015.

93. Murphy SN, Castro V, Colecchi J, *et al.* *Partners HealthCare OMOP Study Report*., 2011.

94. Madigan D, Stang PE, Berlin JA, *et al.* A systematic statistical approach to evaluating evidence from observational studies. *Annual Review of Statistics and Its Application* 2014; **1**: 11–39. doi:10.1146/annurev-statistics-022513-115645.

95. Madigan D, Ryan PB, Schuemie M. Does design matter? Systematic evaluation of the impact of analytical choices on effect estimates in observational studies. *Therapeutic Advances in Drug Safety* 2013; **4**: 53–62. doi:10.1177/2042098613477445.

96. Ji Y, Ying H, Tran J, Dews P, Mansour A, Michael Massanari R. A method for mining infrequent causal associations and its application in finding adverse drug reaction signal pairs. *IEEE Transactions on Knowledge and Data Engineering* 2013; **25**: 721–733. doi:10.1109/TKDE.2012.28.

97. Jin H, Chen J, He H, Kelman C, Mcaullay D, O’keefe CM. Signaling potential adverse drug reactions from administrative health databases. *IEEE Transactions on Knowledge and Data Engineering* 2010; **22**: 839–853. doi:10.1109/TKDE.2009.212.

98. Demailly R. Détection automatisée de signaux en pharmacovigilance chez la femme enceinte à partir de bases médico-administratives. 2021.

99. Chiang C, Penyue Z, Donneyong M, Chen Y, Su Y, Li L. Random Control Selection for Conducting High‐throughput Adverse Drug Events Screening using Large‐scale Longitudinal Health Data. *CPT: Pharmacometrics & Systems Pharmacology* 2021. doi:10.1002/psp4.12673.

100. Støer NC, Botteri E, Thoresen GH, *et al.* Drug use and cancer risk: A drug-wide association study (DWAS) in Norway. *Cancer Epidemiology Biomarkers and Prevention* 2021; **30**: 682–689. doi:10.1158/1055-9965.EPI-20-1028.

101. Huybrechts KF, Kulldorff M, Hernández-Díaz S, *et al.* Active Surveillance of the Safety of Medications Used During Pregnancy. *Am J Epidemiol* 2021; **190**: 1159–1168. doi:10.1093/aje/kwaa288.

102. Friedman GD, Udaltsova N, Chan J, Quesenberry CP, Habel LA. Screening pharmaceuticals for possible carcinogenic effects: Initial positive results for drugs not previously screened. *Cancer Causes and Control* 2009; **20**: 1821–1835. doi:10.1007/s10552-009-9375-2.

103. Patel CJ, Ji J, Sundquist J, Ioannidis JPA, Sundquist K. Systematic assessment of pharmaceutical prescriptions in association with cancer risk: a method to conduct a population-wide medication-wide longitudinal study. *Scientific Reports* 2016; **6**. doi:10.1038/srep31308.

104. Gault N, Castañeda-Sanabria J, de Rycke Y, Guillo S, Foulon S, Tubach F. Self-controlled designs in pharmacoepidemiology involving electronic healthcare databases: a systematic review. *Medical Research Methodology* 2017. doi:10.1186/s12874-016-0278-0.

105. Kulldorff M, Fang Z, Walsh SJ. A Tree-Based Scan Statistic for Database Disease Surveillance. *Biometrics* 2003: 323–331.

106. Tsiropoulos I, Andersen M, Hallas J. Adverse events with use of antiepileptic drugs: A prescription and event symmetry analysis. *Pharmacoepidemiology and Drug Safety* 2009; **18**: 483–491. doi:10.1002/pds.1736.

107. Reps JM, Garibaldi JM, Aickelin U, Soria D, Gibson JE, Hubbard RB. Signalling paediatric side effects using an ensemble of simple study designs. *Drug Safety* 2014; **37**: 163–170. doi:10.1007/s40264-014-0137-z.

108. Kulldorff M, Davis RL, Kolczak M, Lewis E, Lieu T, Platt R. A maximized sequential probability ratio test for drug and vaccine safety surveillance. *Sequential Analysis* 2011; **30**: 58–78. doi:10.1080/07474946.2011.539924.

109. Schuemie MJ. Safety surveillance of longitudinal databases: Further methodological considerations. *Pharmacoepidemiology and Drug Safety* 2012; **21**: 670–672. doi:10.1002/pds.3259.

110. Simpson SE. A Positive Event Dependence Model for Self-Controlled Case Series with Applications in Postmarketing Surveillance. *Biometrics* 2013; **69**: 128–136. doi:10.1111/j.1541-0420.2012.01795.x.

111. Wang S v., Maro JC, Baro E, *et al.* Data Mining for Adverse Drug Events With a Propensity Score-matched Tree-based Scan Statistic. *Epidemiology* 2018; **29**: 895–903. doi:10.1097/EDE.0000000000000907.

112. Lai ECC, Hsieh CY, Yang YHK, Lin SJ. Detecting potential adverse reactions of sulpiride in schizophrenic patients by prescription sequence symmetry analysis. *PLoS ONE* 2014; **9**. doi:10.1371/journal.pone.0089795.

113. Thurin NH, Lassalle R, Schuemie M, *et al.* Empirical assessment of case-based methods for identification of drugs associated with acute liver injury in the French National Healthcare System database (SNDS). *Pharmacoepidemiology and Drug Safety* 2021; **30**: 320–333. doi:10.1002/pds.4983.

114. Thurin NH, Lassalle R, Schuemie M, *et al.* Empirical assessment of case-based methods for identification of drugs associated with upper gastrointestinal bleeding in the French National Healthcare System database (SNDS). *Pharmacoepidemiology and Drug Safety* 2020; **29**: 890–903. doi:10.1002/pds.5038.

115. McDowell RD, Hughes C, Murchie P, Cardwell C. A systematic assessment of the association between frequently prescribed medicines and the risk of common cancers: a series of nested case-control studies. *BMC Medicine* 2021; **19**. doi:10.1186/s12916-020-01891-5.

116. Gruber S, Chakravarty A, Heckbert SR, *et al.* Design and analysis choices for safety surveillance evaluations need to be tuned to the specifics of the hypothesized drug–outcome association. *Pharmacoepidemiology and Drug Safety* 2016; **25**: 973–981. doi:10.1002/pds.4065.

^1–116^
