# Supplementary material for: Methods for drug safety signal detection using routinely collected observational electronic health care data: A systematic review
Source: Pharmacoepidemiol Drug Saf. 2022 Nov 2;32(1):28–43. doi: 10.1002/pds.5548 (PMC10092128; doi:10.1002/pds.5548)
Supplement: Supplementary file 2 — Appendix S1 – Search strategies in the different databases [file PDS-32-28-s002.docx]

**Appendix 1 – Search strategies in the different databases**

1. EMBASE through OVID

| 1 | Electronic Health Record/ |
| --- | --- |
| 2 | Electronic Medical Record/ |
| 3 | (electronic medical record* OR healthcare data* OR longitudinal medical record* OR electronic health record* OR electronic health care record* OR observational data* OR health registr* OR claim* data* OR administrative data* OR real-world data OR real world data OR health plan data* OR routinely collected data OR dispensing data*).mp |
| 4 | Postmarketing surveillance/ |
| 5 | pharmacovigilance/ |
| 6 | (pharmacoepidemiology OR pharmacovigilance OR vaccine vigilance OR vaccinovigilance or drug surveillance OR safety surveillance OR safety monitoring OR drug-outcome association* OR drug safety OR adverse drug OR drug-adverse OR adverse event* OR vaccine’s safety OR vaccine* safety OR statistical alert* OR safety assessment OR unknown effect* OR safety signal*).mp |
| 7 | Adverse drug reaction/si [Side Effect] |
| 8 | Signal Detection/ |
| 9 | Data Mining/ |
| 10 | (signal detection OR signal identification OR pattern discovery OR hypothesis genera* OR signal genera* OR exploratory analysis OR risk identification OR data mining OR screening OR surveillance study).mp |
| 11 | 1 OR 2 OR 3 |
| 12 | 4 OR 5 OR 6 OR 7 |
| 13 | 8 OR 9 OR 10 |
| 14 | 11 AND 12 AND 13 |

1. MEDLINE through OVID

| 1 | Electronic Health Record/ |
| --- | --- |
| 2 | Electronic Medical Record/ |
| 3 | (electronic medical record* OR healthcare data* OR longitudinal medical record* OR electronic health record* OR electronic health care record* OR observational data* OR health registr* OR claim* data* OR administrative data* OR real-world data OR real world data OR health plan data* OR routinely collected data OR dispensing data*).mp |
| 4 | Drug safety side effects and adverse reactions/ |
| 5 | pharmacovigilance/ |
| 6 | (pharmacoepidemiology OR pharmacovigilance OR vaccine vigilance OR vaccinovigilance or drug surveillance OR safety surveillance OR safety monitoring OR drug-outcome association* OR drug safety OR adverse drug OR drug-adverse OR adverse event* OR vaccine’s safety OR vaccine* safety OR statistical alert* OR safety assessment OR unknown effect* OR safety signal*).mp |
| 7 | Product surveillance, postmarketing/ |
| 8 | Data Mining/ |
| 9 | (signal detection OR signal identification OR pattern discovery OR hypothesis genera* OR signal genera* OR exploratory analysis OR risk identification OR data mining OR screening OR surveillance study).mp |
| 11 | 1 OR 2 OR 3 |
| 12 | 4 OR 5 OR 6 OR 7 |
| 13 | 8 OR 9 |
| 14 | 11 AND 12 AND 13 |

1. Pubmed

((electronic medical record*[Title/Abstract]) OR (electronic patient record*[Title/Abstract]) OR (electronic health care record*[Title/Abstract]) OR (healthcare data*[Title/Abstract]) OR (longitudinal medical record*[Title/Abstract]) OR (electronic health record*[Title/Abstract]) OR (observational data*[Title/Abstract]) OR (health registr*[Title/Abstract]) OR (claim* data*[Title/Abstract]) OR (administrative data*[Title/Abstract]) OR (real-world data[Title/Abstract]) OR (real world data[Title/Abstract]) OR (health plan data*[Title/Abstract]) OR (routinely collected data[Title/Abstract]) OR (dispensing data*[Title/Abstract])) AND ((signal detection[Title/Abstract]) OR (pattern discovery[Title/Abstract]) OR (hypothesis genera*[Title/Abstract]) OR (signal identification[Title/Abstract]) OR (risk identification[Title/Abstract]) OR (signal genera*[Title/Abstract]) OR (data mining[Title/Abstract]) OR (screening[Title/Abstract])) AND ((pharmacoepidemiology[Title/Abstract]) OR (pharmacovigilance[Title/Abstract]) OR (vaccine vigilance[Title/Abstract]) OR (vaccinovigilance[Title/Abstract]) OR (drug safety[Title/Abstract]) OR (drug surveillance[Title/Abstract]) OR (safety surveillance[Title/Abstract]) OR (safety monitoring[Title/Abstract]) OR (drug-outcome association*[Title/Abstract]) OR (adverse drug[Title/Abstract]) OR (drug-adverse[Title/Abstract]) OR (adverse event*[Title/Abstract]) OR (vaccine's safety[Title/Abstract]) OR (vaccine* safety[Title/Abstract]) OR (unknown effect*[Title/Abstract]) OR (statistical alert*[Title/Abstract]) OR (safety assessment[Title/Abstract]) OR (safety signal*[Title/Abstract]) )

1. Scopus, Web of Science and Cochrane library

## TITLE-ABS-KEY ( ( "electronic medical Record*"  OR  "electronic patient record*"  OR  "healthcare data*"  OR  "longitudinal medical record*"  OR  "electronic health record*"  OR  "observational data*"  OR  "health registr*"  OR  "claim* data*"  OR  "administrative data*"  OR  "real-world data"  OR  "real world data"  OR  "health plan data*"  OR  "routinely collected data"  OR  "dispensing data*" )  AND  ( "signal detection"  OR  "signal identification"  OR  "risk identification"  OR  "pattern discovery"  OR  "hypothesis genera*"  OR  "signal genera*"  OR  "data mining"  OR  "exploratory analysis"  OR  "screening"  OR  "surveillance study" )  AND  ( "pharmacoepidemiology"  OR  "pharmacovigilance"  OR  "vaccine vigilance"  OR  "vaccinovigilance"  OR  "drug safety"  OR  "drug surveillance"  OR  "safety surveillance"  OR  "safety monitoring"  OR  "drug-outcome association*"  OR  "adverse drug"  OR  "drug-adverse"  OR  "adverse event*"  OR  "unknown effect*"  OR  "statistical alert*"  OR  "safety signal*"  OR  "vaccine's safety"  OR  "vaccine*safety"  OR  "safety assessment" ) )
